# Case report: Ultrasound-guided removal of foreign matter from the chest wall

**DOI:** 10.1016/j.ijscr.2023.109091

**Published:** 2023-11-24

**Authors:** Jing Lin, Huifei Deng, Qiuyan Yang, Youbo Zuo

**Affiliations:** a Department of Anesthesiology, Affiliated Hospital of North Sichuan Medical College, Nanchong 637000, China; b Department of Emergency, Affiliated Hospital of North Sichuan Medical College, Nanchong 637000, China

**Keywords:** Ultrasound-guided, Chest wall, Foreign matter, Ultrasound intervention, Case report

## Abstract

**Introduction and importance:**

Positioning nodule with a steel wire in pulmonary surgery is a common preoperative step. To date, no reports have been published on the retention of steel wires in the body post-surgery, nor have there been studies describing the ultrasound-guided removal of foreign objects from the chest wall. This report describes a case of a foreign matter was removed from the chest wall by ultrasound-guided.

**Case presentation:**

A 70-year-old woman underwent thoracoscope resection of a pulmonary nodule; however, a fragment of the positioning steel wire remained in the chest wall during the surgery. The anesthetist located the residual steel wire using ultrasound, and subsequently, the surgeon successfully removed it.

**Clinical discussion:**

Detection of foreign matter in the body is rare and usually associated with trauma or accidental retention of materials such as absorbable gelatin sponges or sutures during surgery, which are often found using X-rays. This process is often time-consuming, and X-rays being radioactive are potentially harmful to patients and medical workers. Ultrasonic waves are safe and offer a convenient alternative for such procedures. We removed the residual steel wire through a 0.5 cm skin incision, this method neither caused trauma nor increased costs.

**Conclusion:**

Ultrasonography-assisted positioning is a rapid, convenient, and safe technique, promising to enhance future surgical interventions.

## Highlights

• Ultrasound, considered as the third eye of a physician, has an important role in ultrasonic diagnosis.
• Sometimes foreign matter may stay in body while undergo a medical examination, Ultrasound could find them quickly.
• The patient suffer little pain and tiny incision.

## 1 Introduction

Medical materials such as absorbable gelatin sponges or sutures are the most common foreign objects retained in the body [1]. Although uncommon, surgeons occasionally encounter foreign materials resulting from traumatic injuries that necessitate surgical intervention [2,3]. Invasive procedures can lead to significant tissue damage and adversely affect recovery outcomes. Ultrasound plays a critical role in diagnostic imaging and nerve block procedures, augmenting the clinician's diagnostic capabilities [4]. To our knowledge, there are no reported cases of ultrasound-guided excision of foreign objects from the chest wall. Herein, we report a case study detailing the ultrasound-guided removal of a retained steel wire, initially used for lesion localization, from the chest wall. This work has been reported in line with the SCARE 2023 criteria [5].

## 2 Case presentation

**Fig. 1:**
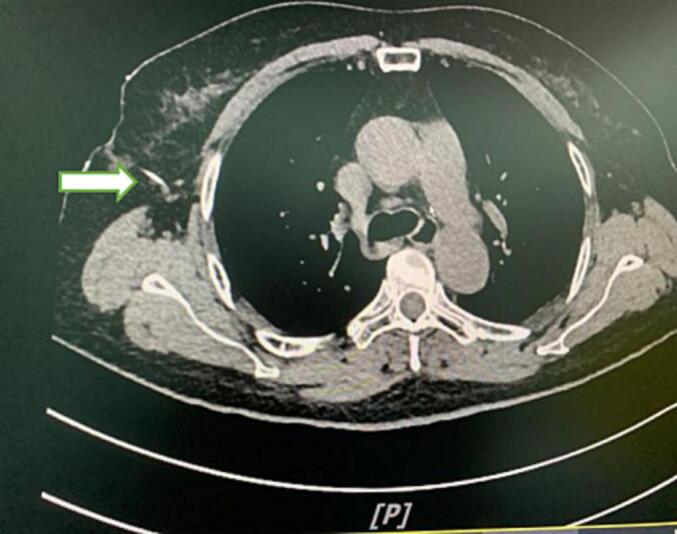
Chest computed tomography displaying the steel wire in the thoracic cavity (arrow).

**Fig. 2:**
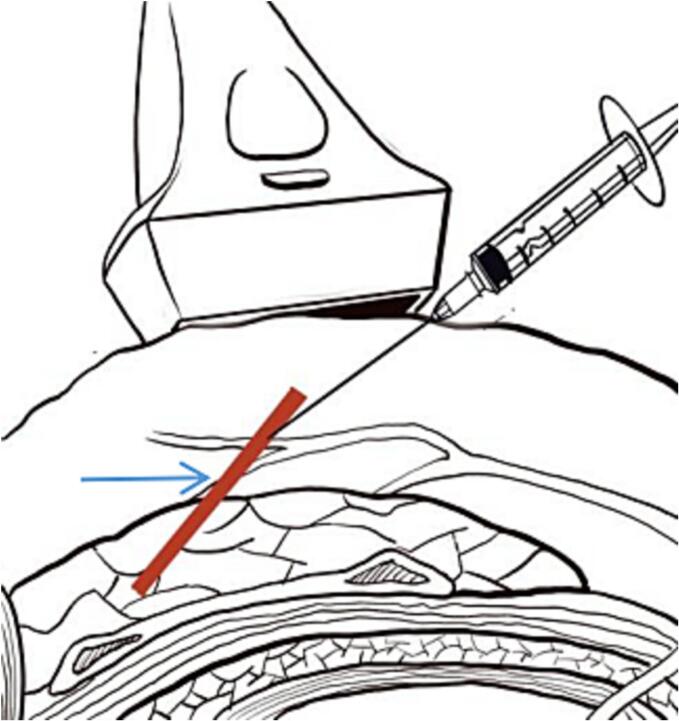
Schematic diagram of ultrasound localization, → steel wire.

**Fig. 3:**
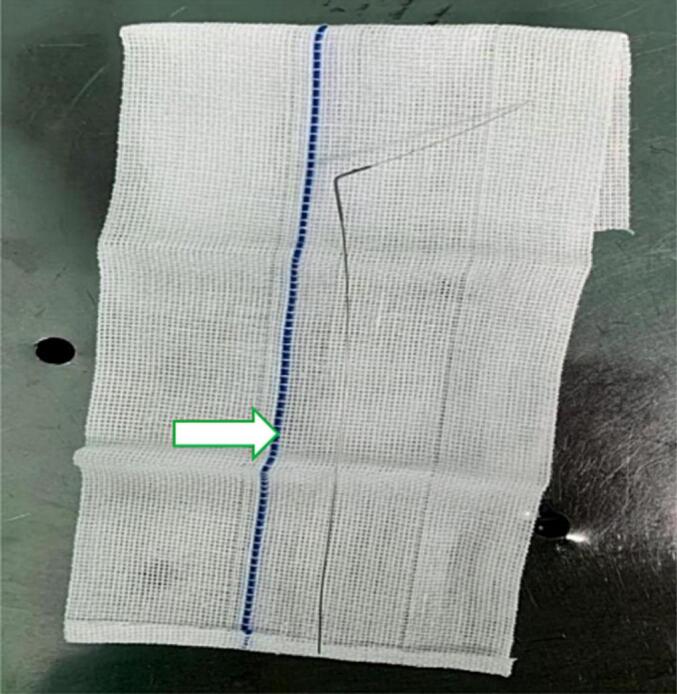
Residual steel wire, the arrow indicates the developed line of the medical gauze.

A 70-year-old woman was diagnosed with a small nodule in the right lung. As the nodule was too small to be pinpointed with a thoracoscope, the patient underwent a computed tomography (CT)-guided percutaneous puncture (Fig. 1) on the day of surgery before entering the operating room. At the beginning of surgery, the thoracic cavity was insufflated with carbon dioxide, in order to avoid the steel wire from affecting the surgical operation, the thoracic surgeon intended to cut the redundant steel wire and pull it out under direct vision. Unfortunately, the hemostatic forceps clamping the end of the steel wire loosened, and due to the elastic retraction of the tissue, a portion of the steel wire remained in the chest wall. The surgeon did not manipulate the residual steel wire or was able to visualize it with the thoracoscope. Attempts to locate the wire with X-ray imaging were unsuccessful for approximately 30 min. The anesthetist suggested using ultrasonography and employed an ultrasound device (UMT-500, Mindray) to trace the puncture pathway. The distorted steel wire quickly appeared within the scanning range. Under ultrasound guidance, a 5 ml injection syringe needle (220,818, Hong Da, Jiang Xi) was used to indicate the location of the steel wire (Fig. 2). The surgeon took out about 14 cm long steel wire through a 0.5 cm skin incision with the guide of the syringe needle (Fig. 3). The entire procedure was successfully completed in approximately 5 min before the surgery commenced. The patient recovered well at the postoperative follow-up.

## 3 Discussion

Foreign matter during surgery is rare, absorbable gelatin sponge or suture are the most common[ [[1], [2], [3]]], Other foreign matters detected include locking plate used for fixing rib fracture or sternum fracture [6]. Xu MY et al. reported a case where horsehair was detected inside the breast of a lactating woman [7].

There are no reports describing the successful ultrasound-guided minimally invasive removal of foreign matter from the chest wall. Ultrasound is primarily utilized for diagnostic purposes and administering nerve blocks before surgery [4]. Cardiac surgery requires monitoring of cardiac valve conditions via ultrasound [8]. Application of ultrasound to other aspects during operation is rare.

During operations, surgeons may accidentally leave sewing needles and gauze inside the body, which are typically detected using X-rays [9]. This process is often time-consuming, and X-rays being radioactive are potentially harmful to patients and medical workers. Ultrasonography is a very safe and convenient modality for intraoperative use. Thibaut Jacques et al. reported that under continuous ultrasound guidance, deep contraceptive implants could be removed in a minimally invasive manner [10].

Under ultrasound imaging, both the fascia tissue and the steel wire appeared hyperechoic [11]. To distinguish between the fascia tissue and the steel wire under ultrasound imaging, we used a 5 ml injection syringe needle as a guide for the steel wire. When the needle tip touched the steel wire, the anesthetist felt a metallic scratching sensation, the 5ml syringe needle was retained here, assisting the anesthetist locating the steel wire, then the surgeon took the steel wire out via 0.5 cm skin incision with the help of the location 5 ml syringe needle.

## 4 Conclusion

We reported a case of ultrasound-guided extraction of a residual steel wire via a minimally invasive 0.5 cm skin incision. This technique resulted in neither trauma nor additional cost. Ultrasonography provided a rapid, convenient, and safe way in this case, thus serving as an alternative choice in future clinical work.

## Funding

The study was funded by the University-level Scientific Research Development Program of North Sichuan Medical College (CBY21-QA48) and the 2023 Scientific Research Development Plan of the Affiliated Hospital of North Sichuan Medical College (2023JC035).

## Patient consent

Written informed consent was obtained from the patient for publication and any accompanying images. A copy of the written consent is available for review by the Editor-in-Chief of this journal on request.

## CRediT authorship contribution statement

Jing Lin and Youbo Zuo generated the experimental hypothesis, designed the study, and wrote the manuscript. Huifei Deng and Qiuyan Yang have been involved in acquisition of data and analyzed the experimental data. All authors read and approved the final manuscript.

## Ethics approval

The study is exempt from ethnical approval in our institution. For the patient consent of this study was obtained from patient, and this did also no damage.

## Registration of research studies

1. Name of the registry: N/A
2. Unique identifying number or registration ID: N/A
3. Hyperlink to your specific registration (must be publicly accessible and will be checked): N/A

## Guarantor

Youbo Zuo is the corresponding author. He is responsibility for the work.

## Declaration of competing interest

All authors declared that it was no conflicts of interest.
